# Supplementary material for: Features of Chromosome Introgression from Gossypium barbadense L. into G. hirsutum L. during the Development of Alien Substitution Lines
Source: Plants (Basel). 2022 Feb 18;11(4):542. doi: 10.3390/plants11040542 (PMC8877206; doi:10.3390/plants11040542)
Supplement: Supplementary file 1 [file plants-11-00542-s001.zip › plants-1546867-supplementary.pdf]

Table 1.

Crossing of aneuploid lines of cotton *G. hirsutum* L. with the line Pima 3-79 of the species *G. barbadense* L.

Chromosome	Crossing variant	Number of bolls obtained		Crossing, %
		Number of crosses	Number of hybrid bolls	
**2**	Mo11 x Pima 3-79	5	3	60,00
	Mo16 x Pima 3-79	12	7	58,33
	Mo19 x Pima 3-79	15	11	73,33
	Mo93 x Pima 3-79	2	1	50,00
**4**	Mo7 x Pima 3-79	2	1	50,00
	Mo31 x Pima 3-79	5	4	80,00
	Mo38 x Pima 3-79	9	6	66,67
	Mo58 x Pima 3-79	4	2	50,00
	Mo59 x Pima 3-79	6	5	83,33
	Mo60 x Pima 3-79	3	3	100,00
	Mo66 x Pima 3-79	20	4	20,00
	Mo69 x Pima 3-79	8	2	25,00
	Mo70 x Pima 3-79	6	4	66,67
	Mo72 x Pima 3-79	12	2	16,66
	Mo73 x Pima 3-79	4	1	25,00
	Mo75 x Pima 3-79	6	4	66,67
	Mo89 x Pima 3-79	4	2	50,00
**6**	Mo13 x Pima 3-79	10	4	40,00
	Mo34 x Pima 3-79	19	5	26,32
	Mo67 x Pima 3-79	5	3	60,00
	Mo92 x Pima 3-79	3	2	66,67
	Mo95 x Pima 3-79	16	8	50,00
**7**	Mo27 x Pima 3-79	2	1	50,00
**12**	Mo94xPima 3-79	3	1	33,33
**17**	Mo56 x Pima 3-79	10	4	40,00
**18**	Mo48 x Pima 3-79	6	1	16,66
**21**	Mo42 x Pima 3-79	6	1	16,67
**22**	Mo17 x Pima 3-79	7	1	14,29
**telo 6**	Telo12xPima 3-79	2	1	50,00
**telo 11**	Mo21 x Pima 3-79	3	3	100,00

Table 2.

Setting of F_0 hybrid seeds obtained from crosses of aneuploid lines of the species *G.hirsutum* L. with the line Pima3-79 of the species *G.barbadense* L.

Chromosome	Crossing variant	Number of bolls	Number of seeds in bolls		Total number of seeds and motes	Setting of hybrid seeds, %
			matured	immature and motes		
-	L-458xPima 3-79	4	79	28	107	73,83±4,25
**2**	Mo11xPima 3-79	3	26	57	83	31,33±5,09
	Mo16xPima 3-79	7	74	135	209	35,41±3,31
	Mo19xPima 3-79	11	152	221	373	40,75±2,54
	Mo93xPima 3-79	1	17	11	28	60,71±10,48
**4**	Mo7xPima 3-79	1	9	13	22	40,91±10,48
	Mo31xPima 3-79	4	56	87	143	39,16±4,08
	Mo38 xPima 3-79	6	79	77	156	50,64±4,00
	Mo58 xPima 3-79	2	23	36	59	38,98±6,35
	Mo59 xPima 3-79	5	79	122	201	39,30±3,45
	Mo60 xPima 3-79	3	31	53	84	36,90±5,27
	Mo66xPima 3-79	4	29	31	60	48,33±6,45
	Mo69 xPima 3-79	2	23	26	49	46,94±7,13
	Mo70xPima 3-79	2	19	54	73	26,03±5,14
	Mo72xPima 3-79	2	41	43	84	51,52±6,15
	Mo73xPima 3-79	1	21	13	34	61,76±8,33
	Mo75 xPima 3-79	4	75	67	142	52,82±4,19
	Mo89xPima 3-79	2	29	31	60	48,33±6,45
**6**	Mo13 xPima 3-79	3	33	60	93	35,48±4,96
	Mo34 xPima 3-79	3	34	68	102	33,33±4,67
	Mo67 xPima 3-79	2	11	48	59	18,64±5,07
	Mo92 xPima 3-79	2	17	43	60	28,33±5,82
	Mo95 xPima 3-79	8	77	219	296	26,01±2,55
**7**	Mo27 xPima 3-79	1	17	26	43	39,53±7,46
**12**	Mo94xPima 3-79	1	19	14	33	57,58±8,60
**17**	Mo56xPima 3-79	4	56	80	136	41,17±4,22
**18**	Mo48 xPima 3-79	1	19	4	23	82,61±7,90
**21**	Mo42xPima 3-79	1	21	13	34	61,76 ±8,33
**22**	Mo17xPima 3-79	1	10	24	34	29,41±7,81
**telo 6**	Telo12xPima 3-79	1	29	13	42	69,05±7,13
**telo 11**	Mo21xPima 3-79	3	12	63	75	16,00±4,23

Table 3.

Germination of F_0 hybrid seeds obtained from crosses of aneuploid lines of the species *G. hirsutum* L. with the line Pima 3-79 of the species *G. barbadense* L.

Chromosome	Crossing variant	Number of seeds sown	Number of shoots	Germination,%
-	F_1 L-458 x Pima 3-79	6	4	66,67
2	F_1 Mo11 x Pima 3-79	17	16	94,12
	F_1 Mo16 x Pima 3-79	8	8	100,00
	F_1 Mo19 x Pima 3-79	14	8	57,14
	F_1 Mo93 x Pima 3-79	15	11	73,33
4	F_1 Mo7 x Pima 3-79	9	7	77,78
	F_1 Mo31 x Pima 3-79	19	4	21.05
	F_1 Mo38 x Pima 3-79	37	28	75,68
	F_1 Mo58 x Pima 3-79	13	7	53,85
	F_1 Mo59 x Pima 3-79	29	18	62,07
	F_1 Mo60 x Pima 3-79	24	18	75,00
	F_1 Mo66 x Pima 3-79	22	8	36,36
	F_1 Mo69 x Pima 3-79	21	11	52,38
	F_1 Mo70x Pima 3-79	19	12	63,16
	F_1 Mo72 x Pima 3-79	20	13	65,00
	F_1 Mo73 x Pima 3-79	21	14	66,67
	F_1 Mo75 x Pima 3-79	15	12	80,00
	F_1 Mo89 x Pima 3-79	29	13	44,83
6	F_1 Mo13 x Pima 3-79	8	8	100
	F_1 Mo34 x Pima 3-79	58	44	75,86
	F_1 Mo67 x Pima 3-79	6	4	66,67
	F_1 Mo92 x Pima 3-79	15	15	100
	F_1 Mo95 x Pima 3-79	42	34	80,95
7	F_1 Mo27 x Pima 3-79	17	15	88,24
12	F_1 Mo94xPima 3-79	19	16	84,21
17	F_1 Mo56xPima 3-79	19	17	89,47
18	F_1 Mo48 xPima 3-79	19	17	89,47
21	F_1 Mo42xPima 3-79	21	13	61,90
22	F_1 Mo17xPima 3-79	10	7	70,00
telo 6	F_1Telo12 x Pima 3-79	28	27	96.43
telo 11	F_1 Mo21 x Pima 3-79	18	16	88,88

Table 4.

Analysis of hybrid F_1 offspring obtained from crosses of aneuploid lines of the species *G. hirsutum* L. with the line Pima 3-79 of the species *G. barbadense* L.

Chromosome	Crossing variant	Total number of hybrid plants in the family	Number of studied plants	Number of disomics (2n=52)	Number of monoso mics (2n=51)	Number of monotelodiso mics (2n=51+I[t])
2	F_1Mo11xPima 3-79					
	765 n	9	2	2	-	
	766 n	7	1	-	1	
	F_1Mo16xPima 3-79					
	98 n	8	6	4	2	
	F_1 Mo19 x Pima 3-79					
	99 n	8	8	8	-	
	768 n	1	1	-	1	
	769 n	6	4	3	1	
	F_1 Mo93 x Pima 3-79					
	516 n	11	9	7	2	
4	F_1Mo7xPima 3-79					
	683 n	7	4	2	2	
	F_1Mo31xPima 3-79					
	770 n	4	2	-	2	
	F_1Mo38xPima 3-79					
	690 n	13	4	3	1	
	F_1Mo58xPima 3-79					
	530 n	7	4	1	3	
	F_1Mo59xPima 3-79					
	531 n	10	5	2	3	
	F_1Mo60xPima 3-79					
	694 n	9	2	1	1	
	F_1Mo69xPima 3-79					
	695 n	7	6	6	-	
	696 n	11	7	6	1	
	F_1Mo70xPima 3-79					
	774 n	9	5	3	2	
	775 n	3	-	-	-	
	F_1Mo75xPima 3-79					
	104 n	12	5	1	4	
	F_1 Mo89 xPima 3-79					
	515 n	8	8	5	3	
	913 n	8	-	-	-	
6	F_1 Mo13 x Pima 3-79					
	97 n	5	3	3	-	
	767 n	2	2	1	1	
	F_1 Mo34 x Pima 3-79					
	2 n	8	6	5	1	
	688 n	9	4	3	1	
	911 n	8	8	7	1	
	F_1 Mo67 x Pima 3-79					
	308 n	4	1	-	1	
	F_1 Mo92 x Pima 3-79					
	539 n	8	7	6	1	
	540 n	6	1	1	-	
	F_1 Mo95 x Pima 3-79					
	105 n	3	2	2	-	
	106 n	8	4	2	2	

	107 n	6	2	2	-	
	108 n	11	7	5	2	
**7**	F_1 Mo27 x Pima 3-79					
	687 n	15	7	6	1	
**12**	F_1 Mo94 x Pima 3-79					
	8 n	16	9	6	3	
**17**	F_1 Mo56 x Pima 3-79					
	4 n	17	17	15	2	
**18**	F_1 Mo48 x Pima 3-79					
	529 n	17	10	9	1	
**21**	F_1Mo42 x Pima 3-79					
	528 n	13	13	12	1	
**22**	F_1 Mo17 x Pima 3-79					
	685 n	7	5	3	2	
**telo 6**	F_1Telo12 x Pima 3-79					
	542 n	24	14	12	-	2
**telo 11**	F_1 Mo21 x Pima 3-79					
	100 n	3	3	2	-	1

Table 5.

Pairing of chromosomes at the metaphase I stage of meiosis in aneuploids F_1 hybrids obtained from crosses of aneuploid lines of the species *G. hirsutum* L. with the line Pima 3-79 of the species *G. barbadense* L.

Chromosome	Crossing variant	Hybrid	Univalent size	Examined cells in MI	Average number per cell		
					univalent	bivalents	quadrivalent
	L-458	-	-	11	-	26,00±0,00	0
	Pima 3-79	-	-	15	-	26,00±0,00	0
	F_1L-458xPima 3-79	680	-	10	-	26,00±0,00	0
2	F_1Mo11xPima 3-79	766_3	Large	8	1,00±0,00	25,00±0,00	0
	F_1Mo16xPima 3-79	98_2	Large	6	1,00±0,00	25,00±0,00	0
		98_6	Large	16	1,00±0,00	25,00±0,00	0
	F_1Mo19xPima 3-79	768_1	Large	11	1,00±0,00	25,00±0,00	0
		769_2	Large	14	1,00±0,00	25,00±0,00	0
	F_1Mo93xPima 3-79	516_2	Large	11	1,00±0,00	25,00±0,00	0
		516_4	Large	14	1,00±0,00	25,00±0,00	0
4	F_1Mo7xPima 3-79	683_1	Medium	20	1,00±0,00	25,00±0,00	0
		683_2	Medium	14	1,00±0,00	25,00±0,00	0
	F_1Mo31xPima 3-79	770_1	Medium	14	1,00±0,00	25,00±0,00	0
		770_2	Medium	11	1,00±0,00	25,00±0,00	0
	F_1Mo38xPima 3-79	690_{11}	Medium	20	1,00±0,00	25,00±0,00	0
		915_7	Medium	5	1,00±0,00	25,00±0,00	0
	F_1Mo58xPima 3-79	530_2	Medium	26	1,00±0,00	25,00±0,00	0
		530_3	Medium	23	1,00±0,00	25,00±0,00	0
		530_7	Medium	5	1,00±0,00	25,00±0,00	0
	F_1Mo59xPima 3-79	531_2	Medium	9	1,00±0,00	25,00±0,00	0
		531_6	Medium	9	1,00±0,00	25,00±0,00	0
		531_8	Medium	6	1,00±0,00	25,00±0,00	0
	F_1Mo60xPima 3-79	694_5	Medium	21	1,00±0,00	25,00±0,00	0
		912_2	Medium	24	1,00±0,00	25,00±0,00	0
	F_1Mo69xPima 3-79	696_9	Medium	14	1,00±0,00	25,00±0,00	0
	F_1Mo70xPima 3-79	774_6	Medium	20	1,00±0,00	25,00±0,00	0
		774_7	Medium	8	1,00±0,00	25,00±0,00	0
	F_1Mo75xPima 3-79	104_2	Medium	5	1,00±0,00	25,00±0,00	0
		104_{11}	Medium	10	1,00±0,00	25,00±0,00	0
	F_1Mo89xPima 3-79	515_2	Medium	14	1,00±0,00	25,00±0,00	0
		515_5	Medium	9	1,00±0,00	25,00±0,00	0
		515_7	Medium	5	1,00±0,00	25,00±0,00	0
6	F_1Mo13xPima 3-79	767_1	Large	17	1,00±0,00	25,00±0,00	0
	F_1Mo34xPima 3-79	2_8	Large	22	1,09±0,16	24,95±0,08	0
		688_9	Large	26	1,00±0,00	25,00±0,00	0
		911_8	Large	15	1,00±0,00	25,00±0,00	0
	F_1Mo67xPima 3-79	308_1	Large	5	1,00±0,00	25,00±0,00	0
	F_1Mo92xPima 3-79	539_5	Large	21	1,00±0,00	25,00±0,00	0
	F_1Mo95xPima 3-79	106_2	Large	7	1,00±0,00	25,00±0,00	0
		106_5	Large	15	1,00±0,00	24,93±0,06	0,07±0,06
		108_3	Large	15	1,00±0,00	25,00±0,00	0
		108_{10}	Large	5	1,00±0,00	25,00±0,00	0

**7**	F_1Mo27xPima 3-79	687_4	Medium	10	1,00±0,00	25,00±0,00	0
**12**	F_1Mo94xPima 3-79	8_1	Large	2	1,00±0,00	25,00±0,00	0
		8_3	Large	28	1,00±0,00	25,00±0,00	0
		8_{13}	Large	13	1,00±0,00	25,00±0,00	0
**17**	F_1Mo56xPima 3-79	4_{13}	Medium-small	9	1,00±0,00	25,00±0,00	0
		4_{17}	Medium-small	9	1,00±0,00	25,00±0,00	0
**18**	F_1Mo48xPima 3-79	529_{16}	Small	22	1,00±0,00	25,00±0,00	0
**21**	F_1Mo42xPima 3-79	528_1	Medium-small	10	1,00±0,00	25,00±0,00	-
**22**	F_1Mo17xPima 3-79	685_3	Medium-small	7	1,00±0,00	25,00±0,00	-
		685_7	Medium-small	18	1,00±0,00	25,00±0,00	-
**telo 6**	F_1Telo12xPima 3-79	542_8	-	3	0	26,00±0,00*	0
		542_{16}	-	4	0	26,00±0,00*	0
**telo 11**	F_1Mo21xPima 3-79	100_1	-	15	0	26,00±0,00*	0
		102_1	-	4	0	26,00±0,00*	0

Note – 25 normal (closed) bivalents and one heteromorphic (open) bivalent.

Table 6.

Analysis of sporades in interspecific F_1 hybrids obtained from crosses of aneuploid lines of the species *G. hirsutum* L. with the line Pima3-79 of the species *G. barbadense* L.

Chromosome	Crossing variant	Hybrid	Total number of microspores	Meiotic index, %	% of tetrads with micronuclei
	L-458	-	1125	99,02±0,29	0,36±0,18
	Pima 3-79	-	1130	98,58±0,35	0,27±0,15
	L-458 x Pima 3-79	680	2535	98,62±0,23	0,04±0,04
**2**	F_1Mo11xPima 3-79	766_3	1567	98,72±0,28	0,51±0,18
	F_1Mo16xPima 3-79	98_2	3524	98,50±0,21	0,57±0,13
**4**	F_1Mo7xPima 3-79	683_1	576	98,07±0,58	1,23±0,46
		683_2	4775	98,35±0,18	0,02±0,02
	F_1Mo31xPima 3-79	770_1	592	96,28±0,78	2,03±0,58
		770_2	1031	96,99±0,53	0,10±0,10
	F_1Mo38xPima 3-79	690_{11}	1182	96,79±0,51	1,18±0,31
	F_1Mo58xPima 3-79	530_2	1023	98,44±0,39	0,10±0,10
		530_3	2902	97,38±0,30	0,41±0,12
		530_7	1764	96,88±0,41	0,68±0,20
	F_1Mo59xPima 3-79	531_2	1476	99,93±0,07	0,14±0,10
		531_6	2904	99,52±0,13	0,07±0,05
		531_8	3694	98,35±0,21	0,14±0,06
	F_1Mo60xPima 3-79	694_5	4768	96,02±0,28	1,15±0,15
	F_1Mo69xPima 3-79	696_9	1469	98,57±0,31	0,00±0,00
	F_1Mo70xPima 3-79	774_6	1435	90,73±0,77	0,00±0,00 **(9,20±0,76 monad)**
		774_7	2167	98,29±0,28	0,05±0,05
	F_1Mo75xPima 3-79	104_2	1639	99,08±0,24	0,06±0,06
		104_{11}	990	99,19±0,28	0,10±0,10
	F_1Mo89xPima 3-79	515_2	2431	98,31±0,26	0,70±0,17
		515_7	1008	97,62±0,48	0,30±0,17
**6**	F_1Mo13xPima 3-79	767_1	5600	98,04±0,19	0,38±0,08
	F_1Mo34xPima 3-79	2_8	1025	97,76±0,46	0,98±0,31
		688_9	2831	97,39±0,30	0,74±0,16
		911_8	1027	97,57±0,48	0,97±0,31
	F_1Mo67xPima 3-79	308_1	1588	95,78±0,50	0,44±0,17
	F_1Mo92xPima 3-79	539_5	1659	98,81±1,19	1,86±0,22
	F_1Mo95xPima 3-79	106_2	1190	99,16±0,26	0,00±0,00
		106_5	986	94,32±0,74	0,81±0,29
**7**	F_1Mo27xPima 3-79	687_4	2348	95,87±0,41	0,38±0,13
**12**	F_1Mo94xPima 3-79	8_1	5196	98,54±0,17	0,27±0,07
		8_3	4272	98,08±0,21	0,33±0,09
		8_{13}	967	98,35±0,41	1,24±0,36
**17**	F_1Mo56xPima 3-79	4_{17}	1015	97,54±0,49	0,99±0,31
**18**	F_1Mo48xPima 3-79	529_{16}	5002	98,14±0,19	0,04±0,03
**21**	F_1Mo42xPima 3-79	528_1	1846	97,67±0,35	0,11±0,08
**22**	F_1Mo17xPima 3-79	685_7	1774	99,27±0,20	0,28±0,13
**telo 6**	F_1Telo12xPima 3-79	542_8	1248	98,56±0,34	0,00±0,00
		542_{16}	4254	98,40±0,19	0,07±0,04
**telo 11**	F_1Mo21xPima 3-79	100_1	4074	98,82±0,17	0,05±0,03

		102_1	1147	95,64±0,60	0,35±0,17

Table 7.

Analysis of pollen fertility in interspecific F_1 hybrids obtained from crosses of aneuploid lines of the species *G. hirsutum* L. with the line Pima 3-79 of the species *G. barbadense* L.

Chromosome	Crossing variant	Hybrd	Total pollen count	Pollen fertility,%	Pollen sterility,%
	L-458	-	628	90,92±1,15	9,08±1,15
	Pima 3-79	-	581	84,34±1,51	15,66±1,51
	F_1 L-458xPima 3-79	680	536	80,78±1,70	19,22±1,70
2	F_1Mo11xPima 3-79	766_3	838	83,41±1,28	16,59±1,28
	F_1Mo16xPima 3-79	98_2	853	79,13±1,39	20,87±1,39
		98_6	526	80,42±1,73	19,58±1,73
	F_1Mo19xPima 3-79	769_2	560	85,71±1,48	14,29±1,48
	F_1Mo93xPima 3-79	516_2	712	82,02±1,44	17,98±1,44
		516_4	224	78,13±2,76	21,88±2,76
4	F_1 Mo7xPima 3-79	683_1	881	90,12±1,01	9,88±1,01
		683_2	920	79,89±1,32	20,11±1,32
	F_1Mo31xPima 3-79	770_1	503	89,07±1,39	10,93±1,39
		770_2	920	89,13±1,03	10,87±1,03
	F_1 Mo38xPima 3-79	690_{11}	936	86,11±19,97	13,89±1,13
	F_1 Mo58xPima 3-79	530_2	1328	83,51±1,02	16,49±1,02
		530_3	1115	84,39±1,09	15,61±1,09
		530_7	319	83,07±2,10	16,93±2,10
	F_1 Mo59xPima 3-79	531_2	899	81,98±1,28	18,02±1,28
		531_6	298	89,93±1,74	10,07±1,74
		531_8	425	72,24±2,17	27,76±2,17
	F_1 Mo60xPima 3-79	694_5	774	75,97±1,54	24,03±1,54
		912_2	633	92,10±1,07	7,90±1,07
	F_1 Mo69xPima 3-79	696_9	925	82,59±1,25	17,41±1,25
	F_1Mo70xPima 3-79	774_6	619	91,44±1,12	8,56±1,12
		774_7	680	87,50±1,27	12,50±1,27
	F_1 Mo75xPima 3-79	104_2	984	80,79±1,26	19,21±1,26
		104_{11}	869	81,24±1,32	18,76±1,32
	F_1Mo89xPima 3-79	515_2	1095	90,96±0,87	9,04±0,87
		515_7	942	90,23±0,97	9,77±0,97
6	F_1Mo13xPima 3-79	767_1	701	88,45±1,21	11,55±1,21
	F_1 Mo34xPima 3-79	2_8	790	87,34±1,18	12,66±1,18
		688_9	955	81,15±1,27	18,85±1,27
		911_8	938	85,18±1,16	14,82±1,16
	F_1 Mo67xPima 3-79	308_1	349	93,12±1,35	6,88±1,35
	F_1 Mo92xPima 3-79	539_5	1242	71,34±1,28	28,66±1,28
	F_1 Mo95xPima 3-79	106_2	-	-	-
		106_5	619	89,98±1,21	10,02±1,21
7	F_1 Mo27xPima 3-79	687_4	672	84,23±1,41	15,77±1,41
12	F_1Mo94xPima 3-79	8_1	866	83,49±1,26	16,51±1,26
		8_3	522	84,87±1,57	15,13±1,57
		8_{13}	693	87,73±1,25	12,27±1,25
17	F_1Mo56xPima 3-79	4_{13}	211	88,63±2,19	11,37±2,19
		4_{17}	521	82,92±1,65	17,08±1,65
18	F_1 Mo48xPima 3-79	529_{16}	777	76,58±1,52	23,42±1,52
21	F_1Mo42xPima 3-79	528_1	1086	82,69±1,15	17,31±1,15
22	F_1Mo17xPima 3-79	685_7	822	81,39±1,36	18,61±1,36

**telo 6**	F_1 Telo12x Pima 3-79	542_8	345	72,17±2,41	27,83±2,41
		542_{16}	763	79,03±1,47	20,97±1,47
**telo 11**	F_1 Mo21xPima 3-79	100_1	553	84,45±1,54	15,55±1,54
		102_1	581	90,53±1,21	9,47±1,21

Table 8.

Crossing of monosomic and monotelodisome lines of cotton with interspecific aneuploid F_1 (Mo x Pima 3-79 or Telo x Pima 3-79) hybrids with substitutions of individual chromosomes or their arms

Chromosome	Hybrids	Number of bolls received (2014–2020)		Crossing, %
		Number of crosses	Number of hybrid bolls	
2	F_1BC_1 (Mo16 x $F_1(98_6)$)	25	6	24,00
	F_1BC_1 (Mo93x $F_1(516_2)$)	3	1	33,33
4	F_1BC_1 (Mo7x $F_1(683_1)$)	3	1	33,33
	F_1BC_1 (Mo31 x $F_1(770_1)$)	2	1	50,00
	F_1BC_1 (Mo38 x $F_1(690_{11})$)	9	2	22,22
	F_1BC_1 (Mo58 x $F_1(530_2)$)	13	2	15,38
	F_1BC_1 (Mo59 x $F_1(531_2)$)	8	1	12,50
	F_1BC_1 (Mo60 x $F_1(694_5)$)	14	9	64,29
	F_1BC_1 (Mo75 x $F_1(104_2)$)	10	6	60,00
6	F_1BC_1 (Mo34 x $F_1(688_9)$)	9	2	22,22
	F_1BC_1 (Mo67 x $F_1(308_1)$)	5	1	20,00
	F_1BC_1 (Mo92 x $F_1(1040_2)$)	13	3	23,08
	F_1BC_1 (Mo95 x $F_1(106_2)$)	4	1	25,00
7	F_1BC_1 (Mo27 x $F_1(687_4)$)	13	7	76,92
12	F_1BC_1 (Mo94 x $F_1(8_1)$)	16	5	31,25
17	F_1BC_1 (Mo56x $F_1(4_{17})$)	8	6	75,00
18	F_1BC_1 (Mo48 x $F_1(529_{16})$	16	2	12,50
21	F_1BC_1 (Mo42 x $F_1(528_1)$	4	3	75,00
22	F_1BC_1 (Mo17 x $F_1(685_7)$)	4	2	50,00
telo 6	F_1BC_1 (Тело12 x F_1 (542_8))	3	1	33,00
telo 11	F_1BC_1 (Mo21x$F_1(100_1)$)	5	4	80,00

Table 9.

Setting of BC_1F_1 hybrid seeds obtained from crosses of monosomic and monotelodisome lines with F_1 aneuploid hybrids with substitutions of specific chromosomes or their arms

Chromosome	Hybrids	Number of bolls	Number of seeds			Total number of seeds and motes	Setting hybrid seeds,%
2	F_1BC_1 (Mo16 x F_1(98$_6$))	6	62	+	85	147	42,18±4,07
	F_1BC_1 (Mo93xF_1(516$_2$))	1	3	+	24	27	11,11±6,05
4	F_1BC_1 (Mo7x F_1(683$_1$))	1	6	+	19	25	24,00±8,54
	F_1BC_1 (Mo31x F_1(770$_1$))	1	7	+	8	15	46,67±12,88
	F_1BC_1 (Mo38xF_1(690$_{11}$))	2	35	+	45	81	44,44±5,52
	F_1BC_1 (Mo58 x F_1(530$_2$))	2	15	+	51	66	22,73±5,16
	F_1BC_1 (Mo59 x F_1(531$_2$))	2	9	+	32	41	21,95±6,46
	F_1BC_1 (Mo60 x F_1(694$_5$))	4	30	+	82	112	24,72±2,64
	F_1BC_1 (Mo75 x F_1(104$_2$))	6	67	+	105	172	38,95±3,72
6	F_1BC_1 (Mo34 x F_1(688$_9$))	2	18	+	48	66	27,27±5,48
	F_1BC_1 (Mo67 x F_1(308$_1$))	1	7	+	16	23	30,43±9,59
	F_1BC_1 (Mo92 xF_1(1040$_2$))	3	8	+	76	84	9,52±3,20
	F_1BC_1 (Mo95 x F_1(106$_2$))	1	3	+	12	15	20,00±10,33
7	F_1BC_1 (Mo27 x F_1(687$_4$))	10	128	+	157	285	44,91±2,95
12	F_1BC_1 (Mo94 x F_1(8$_1$))	5	38	+	109	147	25,85±3,61
17	F_1BC_1 (Mo56x F_1(4$_{17}$))	6	44	+	87	131	33,59±4,13
18	F_1BC_1 (Mo48 x F_1(529$_{16}$)	2	38	+	15	53	71,70±6,19
21	F_1BC_1 (Mo42 x F_1(528$_1$)	3	13	+	47	60	21,67±5,37
22	F_1BC_1 (Mo17 x F_1(685$_7$))	2	10	+	38	48	20,83±5,86
telo 6	F_1BC_1 (Telo12 xF_1(542$_8$))	1	21	+	19	40	52,50±7,90
telo11	F_1BC_1 (Mo21xF_1(100$_1$))	2	5	+	39	44	10,75±3,21

Table 10.

Germination of BC_1F_1 hybrid seeds obtained from crosses of monosomic and monotelodisome lines with aneuploid F_1 (Mo x Pima 3-79 or Telo x Pima 3-79) hybrids with substitutions of specific chromosomes or their arms

Chromosome	Hybrids	Number of hybrid seeds	Number of shoots	Germination, %
**2**	F_1BC_1 (Mo16 x $F_1(98_6)$)	32	17	53,13
	F_1BC_1 (Mo93x$F_1(516_2)$)	3	2	66,67
**4**	F_1BC_1 (Mo7x $F_1(683_1)$)	6	6	100,00
	F_1BC_1 (Mo31x $F_1(770_1)$)	7	6	85,71
	F_1BC_1 (Mo38x$F_1(690_{11})$)	36	16	44,44
	F_1BC_1 (Mo58 x $F_1(530_2)$)	15	11	73,33
	F_1BC_1 (Mo59 x $F_1(531_2)$)	9	7	77,78
	F_1BC_1 (Mo60 x $F_1(694_5)$)	30	17	56,67
	F_1BC_1 (Mo75 x $F_1(104_2)$)	19	10	52,63
**6**	F_1BC_1 (Mo34 x $F_1(688_9)$)	10	10	100,00
	F_1BC_1 (Mo67 x $F_1(308_1)$)	7	4	57,14
	F_1BC_1 (Mo92 x$F_1(1040_2)$)	8	3	37,50
	F_1BC_1 (Mo95 x $F_1(106_2)$)	3	3	100,00
**7**	F_1BC_1 (Mo27 x $F_1(687_4)$)	24	18	75,00
**12**	F_1BC_1 (Mo94 x $F_1(8_1)$)	16	9	56,25
**17**	F_1BC_1 (Mo56x $F_1(4_{17})$)	19	14	73,68
**18**	F_1BC_1 (Mo48 x $F_1(529_{16})$)	38	36	94,74
**21**	F_1BC_1 (Mo42 x $F_1(528_1)$)	13	7	53,85
**22**	F_1BC_1 (Mo17 x $F_1(685_7)$)	10	5	50,00
**telo 6**	F_1BC_1 (Telo12 x$F_1(542_8)$)	21	19	90,48
**telo 11**	F_1BC_1 (Mo21x$F_1(100_1)$)	5	3	60,00

Table 11.

Analysis of BC_1F_1 backcross hybrid offspring obtained from crosses of recurrent parents with aneuploid F_1 (MoxPima 3-79 or Telo x Pima 3-79) hybrids

Chromosome	Crossing variant	Total number of hybrid plants in the family	Number of studied plants	Number of disomics (2n=52)	Number of monoso mics (2n=51)	Number of monotelo disomics (2n=51+I^t)
**2**	$F_1BC_1(Mo16xF_1986_6)$					
	507 n	2	2	2	-	-
	922 n	8	1	-	-	-
	923 n	8	2	1	1	-
	$F_1BC_1(Mo93xF_1516_2)$					
	785 n	2	1	1	-	-
**4**	$F_1BC_1(Mo7xF_1683_2)$					
	508 n	5	5	5	-	-
	$F_1BC_1(Mo31xF_1770_1)$					
	924 n	6	-	-	-	-
	$F_1BC_1(Mo38xF_1690_{11})$					
	294 n	5	5	5	-	-
	925 n	11	1	-	1	-
	$F_1BC_1(Mo58xF_1530_3)$					
	115 n	4	2	1	1	-
	116 n	6	3	3	-	-
	$F_1BC_1(Mo59xF_1531_8)$					
	1041 n	7	7	6	1	-
	$F_1BC_1(Mo60xF_1694_5)$					
	117 n	6	4	2	2	-
	118 n	5	1	1	-	-
	119 n	5	2	2	-	-
	120 n	1	1	1	-	-
	$F_1BC_1(Mo75xF_1104_2)$					
	298 n	8	5	3	2	-
**6**	$F_1BC_1(Mo34xF_1688_9)$					
	293 n	8	4	2	2	-
	$F_1BC_1(Mo67xF_1308_1)$					
	509 n	4	3	3	-	-
	$F_1BC_1(Mo92xF_1539_5)$					
	1040 n	3	3	2	1	-
	$F_1BC_1(Mo95xF_1106_5)$					
	927 n	3	-	-	-	-
**7**	$F_1BC_1(Mo27xF_1687_4)$					
	111 n	8	3	1	2	-
**12**	$F_1BC_1(Mo94xF_18_1)$					
	299 n	7	3	1	2	-
	300 n	2	1	1	-	-
**17**	$F_1BC_1(Mo56xF_14_{17})$					
	512 n	6	5	5	-	-
	513 n	8	6	5	1	-
**18**	$F_1BC_1(Mo48xF_1529_{16})$					
	113 n	14	6	5	1	-
	114 n	20	6	4	2	-
**21**	$F_1BC_1(Mo42xF_1528_1)$					
	781 n	1	-	-	-	-
	782 n	4	2	1	1	-
**22**	$F_1BC_1(Mo17xF_1685_7)$					
	109 n	2	2	2	-	-
	110 n	3	3	1	2	-

**telo 6**	F_1BC_1(Telo12xF_1542_8)					
	561 n	19	11	8	-	3
**telo 11**	F_1BC_1(Mo21xF_1100_1)					
	291 n	2	1	-	-	1
	292 n	1	1	-	-	1

Table 12.

Pairing of chromosomes at the metaphase I stage of meiosis in aneuploid BC_1F_1 hybrids obtained from crosses of recurrent parents with aneuploid F_1 hybrids (Mo x Pima 3-79 or Telo x Pima 3-79)

Chromosome	Crossing variant	Hybrid	Number of examined cells in MI	Univalent size	Average number per cell	
					univalent	Bivalent
	L-458	-	11	-	0	26,00±0,00
	Pima 3-79	-	12	-	0	26,00±0,00
	F_1 (L-458xPima 3-79)	680	10	-	0	26,00±0,00
**2**	F_1BC_1(Mo16x$F_1$98$_6$)	923$_8$	5	Large	1,00±0,00	25,00±0,00
**4**	F_1BC_1(Mo38x$F_1$690$_{11}$)	925$_4$	11	Medium	1,00±0,00	25,00±0,00
	F_1BC_1(Mo58x$F_1$530$_3$)	115$_1$	21	Medium	1,00±0,00	25,00±0,00
	F_1BC_1(Mo59x$F_1$531$_8$)	1041$_4$	24	Medium	1,00±0,00	25,00±0,00
	F_1BC_1(Mo60x$F_1$694$_5$)	117$_4$	14	Medium	1,00±0,00	25,00±0,00
		117$_5$	28	Medium	1,00±0,00	25,00±0,00
	F_1BC_1(Mo75x$F_1$104$_2$)	298$_2$	20	Medium	1,00±0,00	24,67±0,25 (closed)- 0,33±0,25 (open)
		298 $_3$	6	Medium	1,00±0,00	25,00±0,00
**6**	F_1BC_1(Mo34x$F_1$688$_9$)	293$_3$	4	Large	1,00±0,00	25,00±0,00
		293$_7$	4	Large	1,00±0,00	25,00±0,00
	F_1BC_1(Mo92x$F_1$539$_5$)	1040$_2$	11	Large	1,00±0,00	25,00±0,00
**7**	F_1BC_1(Mo27x$F_1$687$_4$)	111$_2$	24	Medium	1,00±0,00	25,00±0,00
		111$_5$	14	Medium	1,00±0,00	25,00±0,00
**12**	F_1BC_1(Mo94x$F_1$8$_1$)	299$_1$	12	Large	1,00±0,00	25,00±0,00
		299$_2$	9	Large	1,00±0,00	25,00±0,00
**17**	F_1BC_1(Mo56x$F_1$4$_{17}$)	513$_8$	6	Medium-small	1,00±0,00	25,00±0,00
**18**	F_1BC_1(Mo48x$F_1$529$_{16}$)	113$_{16}$	5	Small	1,00±0,00	25,00±0,00
		114$_1$	36	Small	1,00±0,00	25,00±0,00
		114$_{20}$	10	Small	1,00±0,00	25,00±0,00
**21**	F_1BC_1(Mo42x$F_1$528$_1$)	782$_1$	5	Medium-small	1,00±0,00	25,00±0,00
**22**	F_1BC_1(Mo17x$F_1$685$_7$)	110$_1$	7	Medium-small	1,00±0,00	25,00±0,00
		110$_3$	8	Medium-small	1,00±0,00	24,25±0,35**
**telo 6**	F_1BC_1(Telo12x $F_1$542$_8$)	561 $_{15}$	21	0	0	26,00±0,00*
		561 $_{17}$	18	0	0	26,00±0,00*
**telo 11**	F_1BC_1(Mo21x $F_1$100$_1$)	291$_1$	17	0	0	26,00±0,00*
		292$_1$	12	0	0	26,00±0,00*

* Note –25 normal (closed) bivalents and one heteromorphic (open) bivalent.

** Note - one univalent plus from 23 to 25 normal (closed) bivalents and one quadrivalent (0.38 ± 0.17 on average per cell).

Table 13.

Analysis of spores in BC_1F_1 hybrids obtained from crosses of recurrent parents with aneuploid F_1 hybrids (Mo x Pima 3-79 or Telo x Pima 3-79)

**Chromosome**	**Crossing variant**	**Hybrid**	**Total number of microspores**	**Meiotic index,%**	**% of tetrads with micronuclei**
	L-458	-	1121	99,38±0,24	0,00±0,00
	Pima 3-79	-	1130	98,58±0,35	0,27±0,15
	F_1L-458 x Pima 3-79	680	2535	98,62±0,23	0,04±0,04
**2**	F_1BC_1(Mo16x$F_1$98$_6$)	923$_8$	-	-	-
	F_1BC_1(Mo38x$F_1$690$_{11}$)	925$_4$	384	95,31±1,08	2,60±0,81
	F_1BC_1(Mo58x$F_1$530$_3$)	115$_1$	5534	95,61±0,28	0,11±0,04
	F_1BC_1(Mo59x $F_1$531$_8$)	1041$_4$	2485	98,99±0,20	0,12±0,07
	F_1BC_1(Mo60x$F_1$694$_5$)	117$_4$	2715	92,90±2,66	0,44±0,19
		117$_5$	1703	95,71±0,49	1,17±0,26
	F_1BC_1(Mo75x $F_1$104$_2$)	298$_2$	1168	96,48±0,54	0,94±0,28
		298$_3$	598	96,49±0,75	1,34±0,47
**6**	F_1BC_1(Mo34x$F_1$688$_9$)	293$_3$	6450	95,72±0,25	0,99±0,12
		293$_7$	5586	98,69±0,15	0,05±0,03
	F_1BC_1(Mo92x$F_1$539$_5$)	1040$_2$	663	89,89±1,17	1,21±0,42
**7**	F_1BC_1(Mo27x$F_1$687$_4$)	111 $_2$	1292	95,43±0,58	0,54±0,20
		111 $_5$	657	96,65±0,70	0,61±0,30
**12**	F_1BC_1(Mo94x$F_1$8$_1$)	299$_1$	-	-	-
		299$_2$	1486	96,37±0,49	1,55±0,32
**17**	F_1BC_1(Mo56x$F_1$4$_{17}$)	513$_8$	-	-	-
**18**	F_1BC_1(Mo48x$F_1$529$_{16}$)	113 $_{16}$	-	-	-
		114 $_1$	2000	97,95±0,32	0,85±0,21
		114 $_{20}$	3170	97,60±0,27	0,06±0,04
**21**	F_1BC_1(Mo42x$F_1$528$_1$)	782$_1$	-	-	-
**22**	F_1BC_1(Mo17x$F_1$685$_7$)	110$_1$	3038	99,37±0,14	0,07±0,05
		110$_3$	2806	98,97±0,19	0,14±0,07
**telo 6**	F_1BC_1(Telo12x$F_1$542$_8$)	561 $_{15}$	2106	98,43±0,27	0,90±0,21
		561 $_{17}$	-	-	-
**telo 11**	F_1BC_1 (Mo21x$F_1$100$_1$)	291 $_1$	548	92,34±1,14	2,55±0,67
		292 $_1$	2191	92,38±0,57	2,46±0,33

Table 14.

Analysis of pollen fertility in aneuploid BC_1F_1 hybrids obtained from crosses of recurrent parents with aneuploid F_1 hybrids (Mo x Pima 3-79 or Telo x Pima 3-79)

Chromosome	Crossing variant	Hybrd	Total pollen count	Pollen fertility,%	Pollen sterility,%
	L-458	-	628	90,92 ± 1,15	9,08 ± 1,15
	Pima 3-79	-	581	84,34 ± 1,51	15,66 ± 1,51
	F_1 L-458 x Pima 3-79	680	536	80,78 ± 1,70	19,22 ± 1,70
**2**	F_1BC_1 (Mo16x$F_1$98$_6$)	923$_8$	-	-	-
**4**	F_1BC_1(Mo38x$F_1$690$_{11}$)	925$_4$	-	-	-
	F_1BC_1(Mo58x$F_1$530$_3$)	115$_1$	1789	91,45 ± 0,66	8,55 ± 0,66
	F_1BC_1(Mo59x$F_1$531$_8$)	1041$_4$	1048	89,79 ± 0,94	10,21 ± 0,94
	F_1BC_1(Mo60x$F_1$694$_5$)	117$_4$	1048	67,27 ± 1,45	32,73 ± 1,45
		117$_5$	715	78,60 ± 1,53	21,40 ± 1,53
	F_1BC_1(Mo75x$F_1$104$_2$)	298$_2$	576	84,38 ± 1,51	15,63 ± 1,51
		298$_3$	-	-	-
**6**	F_1BC_1(Mo34x$F_1$688$_9$)	293$_3$	1019	84,89 ± 1,12	15,1 ± 1,12
		293$_7$	480	90,63 ± 1,33	9,38 ± 1,33
	F_1BC_1(Mo92x$F_1$539$_5$)	1040$_2$	1341	81,80 ± 1,05	18,20 ± 1,05
**7**	F_1BC_1(Mo27x$F_1$687$_4$)	111$_2$	538	80,30 ± 1,71	19,70 ± 1,71
		111$_5$	572	83,57 ± 1,55	16,43 ± 1,55
**12**	F_1BC_1 (Mo94x$F_1$8$_1$)	299$_1$	719	96,38±0,70	3,62±0,70
		299$_2$	640	95,63±0,81	4,38±0,81
**17**	F_1BC_1 (Mo56x$F_1$4$_{17}$)	513$_8$	669	90,43±1,14	9,57±1,14
**18**	F_1BC_1(Mo48x$F_1$529$_{16}$)	113$_{16}$	-	-	-
		114$_1$	687	81,51 ± 1,48	18,49 ± 1,48
		114$_{20}$	491	95,72 ± 0,91	4,28 ± 0,91
**21**	F_1BC_1(Mo42x$F_1$528$_1$)	782$_1$	-	-	-
**22**	F_1BC_1(Mo17x$F_1$685$_7$)	110$_1$	680	72,35±1,72	27,65±1,72
		110$_3$	1510	82,38±0,98	17,62±0,98
**telo 6**	F_1BC_1(Telo12x$F_1$542$_8$)	561 $_{15}$	655	85,65±1,37	14,35±1,37
		561$_{17}$	-	-	-
**telo 11**	F_1BC_1(Mo21x$F_1$100$_1$)	291$_1$	661	89,41±1,20	10,59±1,20
		292$_1$	453	91,61 ±1,30	8,39 ± 1,30
